# Inferring statistical properties of 3D cell geometry from 2D slices

**DOI:** 10.1371/journal.pone.0209892

**Published:** 2019-02-01

**Authors:** Tristan A. Sharp, Matthias Merkel, M. Lisa Manning, Andrea J. Liu

**Affiliations:** 1 Dept. of Physics and Astronomy, University of Pennsylvania, Philadelphia, PA, United States of America; 2 Physics Department, Syracuse University, Syracuse, NY, United States of America; 3 Syracuse Biomaterials Institute, Syracuse, NY, United States of America; Mathematical Institute and the Institue for Biology, Leiden, NETHERLANDS

## Abstract

Although cell shape can reflect the mechanical and biochemical properties of the cell and its environment, quantification of 3D cell shapes within 3D tissues remains difficult, typically requiring digital reconstruction from a stack of 2D images. We investigate a simple alternative technique to extract information about the 3D shapes of cells in a tissue; this technique connects the ensemble of 3D shapes in the tissue with the distribution of 2D shapes observed in independent 2D slices. Using cell vertex model geometries, we find that the distribution of 2D shapes allows clear determination of the mean value of a 3D shape index. We analyze the errors that may arise in practice in the estimation of the mean 3D shape index from 2D imagery and find that typically only a few dozen cells in 2D imagery are required to reduce uncertainty below 2%. Even though we developed the method for isotropic animal tissues, we demonstrate it on an anisotropic plant tissue. This framework could also be naturally extended to estimate additional 3D geometric features and quantify their uncertainty in other materials.

## Introduction

Over the past decade, improved live-imaging techniques including multi-photon confocal [1] and light sheet microscopy [2] have dramatically altered our ability to quantify tissue architecture in *in vivo* and *in vitro* biological systems. In tandem, there has been an increased focus on developing mathematical models that can help organize and drive hypotheses about these complex systems.

Quite a bit of analysis and modeling has focused on confluent monolayers, where there are no gaps or overlaps between cells. These two-dimensional sheets of tissue are often studied in cell culture systems [3–5] and can also be found during embryonic development [6, 7]. Much of that work focuses on understanding how cellular properties (interfacial tensions, adhesion, adherens junctions) give rise to local cellular shapes and also how they help to generate the large-scale, emergent mechanical properties of tissue.

For example, researchers have developed a suite of mechanical inference techniques to estimate interfacial tensions and pressures from detailed images of cell shapes [6, 8, 9]. Others have quantified precisely the deformation mechanisms in the developing fruit fly using dynamical shape changes [10]. These methods rely heavily on automated watershed algorithms to segment membrane-labeled cell images in order to identify cell-cell interfaces in a network of many cells [11–16]. Existing segmentation algorithms have largely been optimized to work on two-dimensional cell sheets.

Another set of experiments and models has focused on the statistics of cell shapes as a metric to quantify global mechanical tissue properties. Specifically, studies of 2D cell vertex models (VMs) have found that cell shape may determine mechanical properties of confluent tissues (tissues with no gaps between cells) [17–19]. The models predict that when cells have a compact shape, so that their cross-sectional perimeter is small relative to their cross-sectional area, the tissue as a whole is solid-like in the sense that cells cannot migrate. In contrast, when cells have an elongated shape, so that their perimeter is large relative to their area, then the tissue is fluid-like in the sense that cells can easily exchange neighbors and migrate. The transition from solid-like to fluid-like behavior is predicted to occur at a specific value of the dimensionless 2D shape index, *p*^2*D*^, which is defined as the ratio of the perimeter to the square root of the area. This prediction was shown to be precisely realized in human epithelial lung cell culture [5].

Given that many biological tissues are fully three dimensional, it is natural to wonder whether any of this work can be extended to 3D. From a modeling perspective, it is straightforward, although technically challenging, to develop 3D simulations. We have recently developed a 3D vertex-like model, called the 3D Voronoi model, and demonstrated that it, too, has a fluid-to-solid transition governed by cell shape [20]. In this case, the governing shape parameter is *p*^3*D*^, which is the dimensionless ratio of the surface area of each cell *S* to its volume *V*: *p*^3*D*^ = *S*/*V*^2/3^. It also appears fairly straightforward to generalize mechanical inference methods to 3D [21].

Although advances in imaging techniques have allowed much clearer and deeper imaging of 3D structures, it remains a technically difficult challenge to reconstruct the full network of cellular contacts in 3D [22–25]. For example, watershed algorithms for segmentation will fail if there is even one 2D slice where the membrane structure is poorly resolved, and so in general they have a very large error rate in 3D. In addition, many 3D structures of interest lie deep inside tissues where optical scattering makes live-imaging techniques difficult. In some cases, such as histological sections for staging of cancer tumors, only individual 2D images are available. Finally, a 3D reconstruction requires that all of the cells must remain sufficiently stationary while an image stack is acquired. Therefore, to our knowledge, very little of the exciting work in 2D can be robustly extended into live-imaged 3D experimental data.

This suggests that there may be an unexplored opportunity to use *statistics* of 2D images, which are standard in the field, to infer something about the *statistics* of 3D structures, an idea which has been exploited previously in materials science. Methods to estimate the grain size distribution within poly-crystalline materials have been proposed that use processed 2D imagery and assume 3D grain shapes [26–28]. Statistical reconstruction of 3D structure from 2D imagery has also been investigated for porous two-phase random media [29], particulate media [30], and media with shaped inclusions [31]. Typically, these methods start with a random 3D structure and have a process for evolving that structure to reduce differences between its 2D projections and 2D experimental data.

In our case, we would like to understand whether we can infer useful 3D shape information from 2D slices. Such an approach will not be directly helpful for mechanical inference methods, which rely on precise reconstructions of angles between junctions in 3D. However, it could prove very useful for testing predictions of vertex-like models where tissue mechanics is predicted to depend on cell shape, or perhaps for testing models for studying constrained cell migration through complex networks. Such migration can lead to DNA damage that depends sensitively on the shapes and sizes of pores in the constraining environment [32].

Therefore, the goal of this manuscript is to test whether information about 3D cell shapes can be reconstructed from randomly selected 2D image slices. Many experiments on mechanics and migration of cells in 3D focus on prepared tissues in collagen matrix or in centrifuged cell aggregates, and on other tissues, including organoids, certain tumors, and certain embryonic tissues, which appear isotropic and have relatively simple structure. We therefore perform this analysis in the context of a 3D Voronoi model [20], which is perhaps the simplest model for confluent cell bodies in 3D. In contrast to previous 3D inference methods, which typically sample only a fraction of the model phase space using Monte Carlo or dynamic minimization techniques [29–31], the simplicity of this model allows us to quantify the relationship between the generated 2D and 3D data across the entire relevant phase space.

We focus on determining whether the mean 3D shape index, which models suggest is strongly correlated with tissue mechanics, can be inferred from the 2D shape index, although we also explore other 2D and 3D shape descriptors as well. We find that there is a robust correlation between the 2D and 3D shape, and quantify the sensitivity of this correlation to sample size, experimentally relevant systematic errors, and tissue heterogeneity. We find that relatively few cells are required to converge on the correct mean 3D cell shape index, and that the estimates are quite robust with respect to moderate errors in 2D cell perimeter measurements, dropping cells with a small cross-sectional area, and cell size heterogeneity. This general framework may be extended to other contexts, ranging from more complicated or anisotropic tissues to extracellular matrix to disordered inorganic materials; all that is required is to substitute the 3D Voronoi model with reasonable 3D models of such systems or full 3D cell shape reconstructions and to replace *p*^3*D*^ with the relevant 3D shape descriptors.

## Results

### 2D cell shapes in slices of 3D cell packings

**Fig 1:**
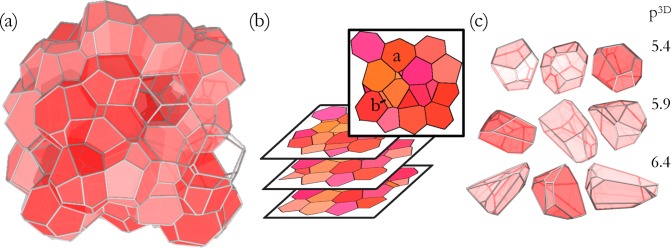
Visualization of the simulated tissue, confocal cross-sections, and individual 3D cell shapes. (a) A 3D tissue geometry in which all 3D cells have the same shape index, *p*^3*D*^ = 5.40, from the 3D Voronoi model. (b) The geometry seen in simulated ideal confocal imagery of that tissue. (c) Samples of the cell shapes from the model with specified *p*^3*D*^ value. A 3D tissue geometry created using the 3D Voronoi model and the geometry seen in simulated ideal confocal imagery. In this example, all 3D cells have the same shape index (*p*^3*D*^ = 5.45).

As described in the methods section, we use the 3D Voronoi model to create cell packings with a specified, homogeneous 3D shape index, given by *p*^3*D*^ = *S*/*V*^2/3^. We cut the packings to obtain parallel slices of randomly oriented systems, yielding 2D networks of edges and vertices, as illustrated in Fig 1. For simplicity, we assume the slices to be extremely thin, although we can also vary the thickness of the optical section as described later. Our systems are small compared to experimentally-obtainable slices; for a large slice of an isotropic tissue, the results would be the same if one analyzed many different cells from the same slice. We then calculate the 2D shape index *p*^2*D*^ for each cell in the 2D slices.

**Fig 2:**
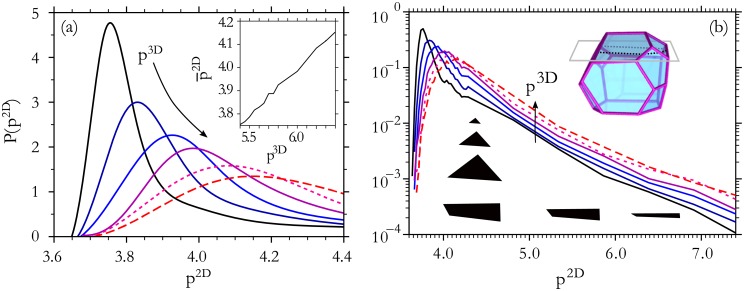
2D cell shape index distributions. (a) The distribution of 2D shape index, *p*^2*D*^, in slices from 3D cell packings provide a signature of the 3D shape index, *p*^3*D*^. *p*^3*D*^ values vary from 5.4 (solid black) to 6.4 (dashed red) in increments of 0.2. (Data available online [33].) (Inset) The location of the distribution peak varies smoothly with *p*^3*D*^. (b) The *p*^2*D*^-distribution tail decays with a similar form for all values of *p*^3*D*^. Example cross sections illustrate that arbitrarily large *p*^2*D*^ values can be produced when the cross section is nearly parallel to a nearby edge—this produces nearly-rectangular slim shapes, for which *p*^2*D*^ diverges when the cutting plane is near the edge. In contrast, general cross sections near a vertex are triangles, and the shape index, which is insensitive to the size of the triangle, retains a constant value independent of proximity of the cutting plane and vertex.

The distributions of *p*^2*D*^ values for 20, 000 cells extracted from 500 3D packings are shown in Fig 2, for several different values of *p*^3*D*^. Each curve demonstrates that even though each of the 3D cells has an identical 3D shape index, *p*^2*D*^ exhibits a broad distribution of values. The lowest possible value of *p*^2*D*^ is that of a circle, $p_{circ}^{2D}=2\sqrt{\pi }\approx 3.54$, although compact shapes in tessellated patterns are typically higher. Fig 1 shows a compact cell (labeled “a”) with a shape index of *p*^2*D*^ = 3.75. In contrast, very elongated shapes occasionally arise when polyhedral edges are nearly parallel to the slicing plane, as shown by the cell labeled “b” in Fig 1. These very elongated shapes are a purely geometric effect that contain little information about the underlying 3D shape index, as shown in Fig 2(b). They contribute to a long tail in the distribution of *p*^2*D*^ that strongly affects both the mean and variance. Therefore, we choose to focus on quantities such as the location of the peak of the distribution, ${\bar{p}}^{2D}$, and the half-width at half max (HWHM) that are less sensitive to these tails.

A first observation is that the peak in the 2D shape index (${\bar{p}}^{2D}$) shifts dramatically with *p*^3*D*^, suggesting that it should be possible to infer the 3D shape index from 2D data. This is quantified by the inset to Fig 2(a), which indicates that ${\bar{p}}^{2D}$ scales linearly with *p*^3*D*^. More comprehensive measures of sensitivity such as the K-S test, discussed below, confirm that the *p*^2*D*^-distribution sensitively reflects the *p*^3*D*^ value.

While vertex models suggest *p*^3*D*^ is an important parameter governing tissue rheology, this approach could also be useful for inferring quantities that are important for other models. Similarly, one might wonder whether there are better 2D shape descriptors than *p*^2*D*^. In a later section, we study alternative geometry descriptors and find similar sensitivity, meaning that the inference can be made as easily. Therefore in the following sections we focus on the shape indices in 2D and 3D (*p*^2*D*^ and *p*^3*D*^), since they are simple to calculate and they control tissue rigidity in 2D and 3D cell vertex models.

### Precision of *p*^3*D*^ estimation depends on sample size

We ask how our estimate of *p*^3*D*^ depends on the sample size (the number of *p*^2*D*^ values extracted from cells in the 2D imagery, *N*). In experiments on 2D lung epithelia [5], the mean shape index of cells only varies about 4% in total. Therefore, the uncertainty in *p*^3*D*^ must be smaller than a few percent.

It can be arduous to obtain the *p*^2*D*^ values upon which the *p*^3*D*^ estimate is based. In some biology experiments, cell boundaries are digitized by an analyst who hand-traces the outlines of labeled membrane proteins on a computer. Alternatively, automated computer algorithms may segment a 2D image into cell (and non-cell) regions, with trade-offs between speed and accuracy, to record the 2D shapes [12, 14, 15]. It is therefore important to establish how many cells must be analyzed to achieve the necessary level of accuracy in the estimate of *p*^3*D*^.

To study how the best estimate of *p*^3*D*^ and its uncertainty depends on the sample size, for each fixed value of *p*^3*D*^ we segment a very large number of 2D cell images (30, 000 cells) and generate a fixed reference distribution for *p*^2*D*^. We repeat this process for 20 values of *p*^3*D*^ spaced equally between 5.4 and 6.4, to generate a library of reference distributions. These reference distributions are publicly available [33].

**Fig 3:**
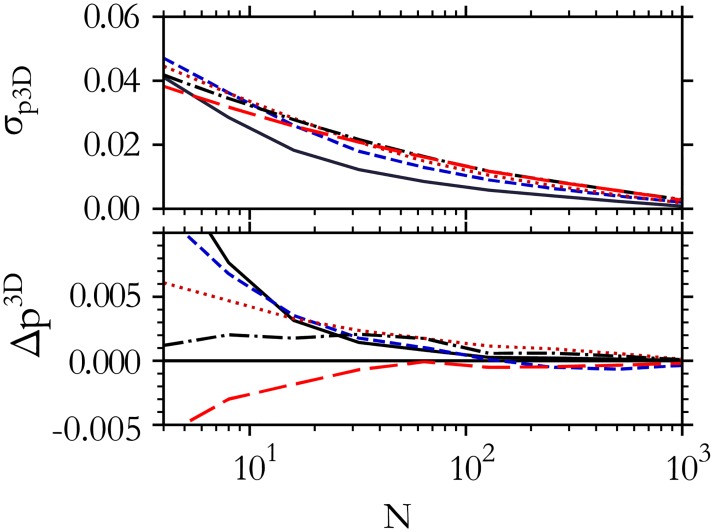
Required number of measured cell shapes. The random error, $\sigma_{p^{3D}}$ (upper), and the systematic error, Δ*p*^3*D*^ (lower), in the *p*^3*D*^ estimate both fall rapidly with the number of cells traced, *N*. The systematic error is much smaller than the random error. The total error can be reduced to 1% by calculating the shape index of about 100 cells in this idealized case where the 2D measurements are exact. Curves are for *p*^3*D*^ = 5.5 (black solid), 5.8 (blue dashed), 6.0 (red dotted), 6.1 (black dash-dotted), 6.2 (red long dashed).

Next, for a fixed value of *p*^3*D*^ we segment *N* randomly selected cells in 2D slices and generate a histogram of *p*^2*D*^. This histogram can be compared to the reference library. The standard Kolmogorov-Smirnov (K-S) test (described in the section below), identifies the most likely distribution that produced the histogram, and correspondingly the estimated *p*^3*D*^ value, $p_{est}^{3D}$. For this purpose we also created a publicly available online tool to compare data to the reference distributions [33]. We repeat this process 1000 times to measure the spread of $p_{est}^{3D}$ estimates that occur. The fractional random error of the *p*^3*D*^ estimate is $\sigma_{p^{3D}}=\sigma_{p_{est}^{3D}}/p^{3D}$, where $\sigma_{p_{est}^{3D}}$ is the standard deviation of $p_{est}^{3D}$. The fractional systematic error is $\Delta p^{3D}=\left(p_{est}^{3D}-p^{3D}\right)/p^{3D}$. Fig 3 shows the random and systematic error as a function of number of cells traced. Note the difference of y-axis scale; Δ*p*^3*D*^ is usually insignificant compared to $\sigma_{p^{3D}}$. After tracing 50 cells at random, the random error in the *p*^3*D*^ estimate is less than 2%. The estimate converges to the actual *p*^3*D*^ with 1% uncertainty after 100 random samples for *p*^3*D*^ = 5.4 and after 200 random samples for *p*^3*D*^ = 5.8. These results show that one may infer a sufficiently accurate estimate of *p*^3*D*^ after imaging only a moderate number of cells.

### Estimation of *p*^3*D*^ is sensitive to systematic tracing errors

For the analysis in the previous section, we assumed that the 2D measurements of cell shape were exact, but data from experiments will have additional sources of noise and error. Therefore, we identify several likely sources and assess their impact on 3D shape estimation.

Cell shapes in 2D imagery can be measured by manual tracing on a computer or by automatic image segmentation and analysis. Some programs that measure cell shape report the perimeter length as the number of pixels that the cell perimeter passes through, and this artificially raises the length of a line segment by an amount that depends on the angle relative to the pixel axes. Attempting to infer the shape of isolated cells rather than a compact cluster of cells that tessellates space can also artificially inflate cell perimeters. Imprecise tracing of shape may result from other factors including inconsistent fluorescent dye saturation, uncertainty in identifying the cell borders, or limited image resolution, for example. We can model these sources of error by distorting the images before processing them and then estimating *p*^3*D*^.

For certain types of noise, the measured distributions of *p*^2*D*^ can be directly computed from those without noise. For example, a systematic overestimate of perimeter by a fraction Δ*L* has the effect of scaling the distributions of Fig 2(a) from *P*(*p*^2*D*^) to *P*(*p*^2*D*^/(1 + Δ*L*))/(1 + Δ*L*). A random mis-estimate of perimeter *L* by a fraction *σ*_*L*_ is modeled by multiplying the perimeter by a Gaussian random factor (with standard deviation *Lσ*_*L*_). This convolves the distributions of Fig 2(a) with a Gaussian kernel.

**Fig 4:**
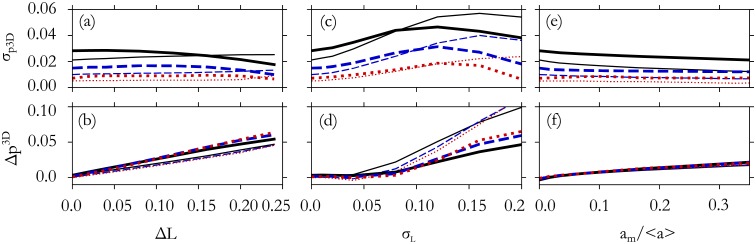
Error propagation in estimate of *p*^3*D*^. The fractional random error, $\sigma_{p^{3D}}$, and the systematic error, Δ*p*^3*D*^, in the *p*^3*D*^ estimate increase with mis-measurement of cell perimeter, but are not significantly influenced by neglecting small cell areas. Curves are for N = 16 (solid), 64 (dashed), and 256 (dotted) cells, and *p*^3*D*^ = 5.6 (thick lines) and *p*^3*D*^ = 6.0 (thin lines). There is little sensitivity to *p*^3*D*^, so results for other *p*^3*D*^ values are not shown. (a,b) A systematic overestimate of cell perimeter by a fraction Δ*L* produces a nearly-proportional systematic error in *p*^3*D*^ estimate. Tracing additional cells reduces uncertainty but does not reduce systematic error. (c,d) A fractional random error of standard deviation *σ*_*L*_ in the cell perimeter measurement also produces an error in the *p*^3*D*^ estimate. (e,f) Accidentally neglecting cells with small area causes only a small error.

Using this information, we can, as in the previous section, compute the errors in the estimated *p*^3*D*^, plotted in Fig 4. Fig 4(a) shows that $\sigma_{p^{3D}}$, the random error in the *p*^3*D*^ estimate, is nearly independent of the perimeter overestimation Δ*L* and decreases with the number of traced cells, to less than 1% with N = 256 cells. Δ*p*^3*D*^, the systematic error, is approximately linear in Δ*L* and the error in the estimate can be reduced below 1% if the perimeter error is less than 3%.

Fig 4(c) and 4(d) show the error accrued from random tracing errors that result in mis-estimate of cell perimeter by a fraction *σ*_*L*_. For large values *σ*_*L*_, the estimated *p*^3*D*^ can differ systematically from the true *p*^3*D*^, but if *σ*_*L*_ < 0.06 (less than 6% uncertainty of each *L* measurement) the systematic error is less than 1%, and tracing a large number of cells reduces the random error in $p_{est}^{3D}$.

Another possible source of systematic error is that the smallest shapes in microscopy may be accidentally overlooked and not traced. We generate reference distributions for this error by filtering the small-area shapes from the distribution functions. Neglecting shapes of area below a certain threshold, *a*_*m*_, introduces relatively little error into the *p*^3*D*^ estimate as shown in Fig 4. When cells with area 10% of the mean are accidentally neglected, the result is only an error of 1% in *p*^3*D*^. To avoid errors when comparing against the distributions generated here, cell shapes of all sizes should be included. If only large cell shapes can be traced reliably in experiment, then the resulting histograms may be compared to reference distributions that are generated using only the large shapes.

The above results show that generally an error in a tracing measurement generates a similar order of magnitude fractional error in the *p*^3*D*^ estimate. If multiple errors occur (*e*.*g*. both Δ*L* and *σ*_*L*_ are significant), and they are independent and uncorrelated, systematic errors Δ*p*^3*D*^ are approximately summed while random errors $\sigma_{p^{3D}}$ are approximately added in quadrature.

### Effect of heterogeneity

So far, we considered homogeneous tissues where all cells have the same cell volume *V* and 3D shape descriptor *p*^3*D*^. Of course, this is an idealization, as in real tissues there will be variations in these quantities. Now, we study the influence of such variations by generating 3D cell packings with Gaussian distributions of shapes *p*^3*D*^ or volumes *V*.

We first focus on variations in cell shape. We again generate the cell packings for an additional 200,000 cells as energy-minimized states of the 3D Voronoi model (see Methods), where states are included only if the cells achieve the target mean $\mu_{p^{3D}}$ and standard deviation $\sigma_{p^{3D}}$ of the 3D shape index; cases near extreme values of $\mu_{p^{3D}}$ (near 5.4 or 6.4) targeting high-$\sigma_{p^{3D}}$ were not able to achieve the targets as seen in other simulations [19, 20] and so are excluded.

**Fig 5:**
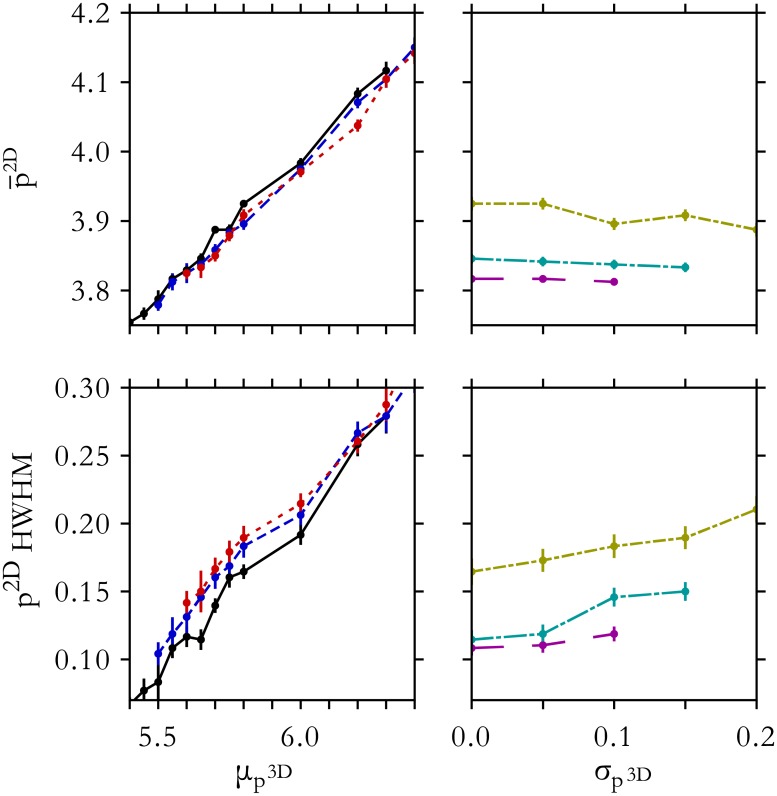
Properties of *p*^2*D*^ distributions from heterogeneous tissues. The peak and half-width-at-half-max (HWHM) of the *p*^2*D*^-distribution accurately reflect the mean constituent 3D cell shape index, but are relatively insensitive to variation in shape. Curves vary $\mu_{p}^{3D}$ for $\sigma_{p^{3D}}=0.0$ (black), 0.1 (blue, dashed), and 0.15 (red, dotted), and vary $\sigma_{p^{3D}}$ for $\mu_{p}^{3D}=5.55$ (magenta, long dash), 5.65 (cyan, long dash-dot), and 5.8 (yellow, dash-dot).

As discussed earlier, we focus on the location of the peak in the 2D shape index (${\bar{p}}^{2D}$) and the half-width-at-half-max (HWHM) to minimize contributions of the universal tail. Fig 5 shows ${\bar{p}}^{2D}$ and HWHM for various values of $\mu_{p^{3D}}$ and $\sigma_{p^{3D}}$. While the peak and width of the distribution are strongly correlated with $\mu_{p^{3D}}$, these quantities are much less sensitive to $\sigma_{p^{3D}}$. A sensitivity analysis based on the K-S test confirms this result for both these and other shape descriptors.

**Fig 6:**
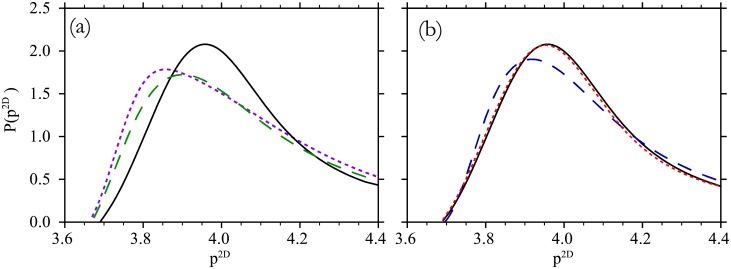
*p*^2*D*^-distributions for heterogeneous 3D cell shape or volume. (a) The *p*^2*D*^-distribution for cell packings of homogeneous 3D shape index *p*^3*D*^ = 5.9 (solid black) is compared to a packing of a binary mixture (dashed green). The binary mixture is 50% of *p*^3*D*^ = 5.6 and 50% of *p*^3*D*^ = 6.2 cells, so that the mean 3D shape index is 〈*p*^3*D*^〉 = 5.9. The superposition (dotted violet) of the *p*^2*D*^-distributions of packings with homogeneous *p*^3*D*^ = 5.6 and *p*^3*D*^ = 6.2, respectively, are a close approximation of the heterogeneous system. (b) The *p*^2*D*^-distribution for cell packings of homogeneous 3D shape index *p*^3*D*^ = 5.9 (solid black) and of heterogeneity in 3D shape index $\sigma_{p^{3D}}=0.15$ (dashed blue) and in cell volume *σ*_*V*_ = 0.15*V*_0_ (dotted red).

It is natural to ask *why and how* the 2D shape distribution is mostly insensitive to the variance in 3D shape index. To gain an intuition for this observation, it is useful to look at a specific example. Fig 6(a) illustrates the 2D shape distribution for a binary mixture of approximately 50% *p*^3*D*^ = 5.6 and 50% *p*^3*D*^ = 6.2 cells. The distribution *P* from the mixed system (dashed green line) is distinct from *P* of the homogeneous systems, the most similar of which is *p*^3*D*^ = 5.9 (solid black line). This emphasizes that the 2D shape distribution from a mixed system is distinct from any of the homogeneous-system distributions.

Next, we plotted the superposition of the homogeneous distributions, *P*_*mix*_ ≈ 0.5*P*_5.45_ + 0.5*P*_6.0_ (Fig 6(a), violet dotted line) and found good agreement with the distribution of the mixed system. This suggests that the mixed system is composed of approximately the same 3D shapes that are generated in homogeneous simulations of the constituent *p*^3*D*^ values. Under this approximation, the *p*^2*D*^-distribution of the heterogeneous system is

$$\begin{array}{c} P_{mix}=\sum_{\beta }f_{\beta }P_{p_{\beta }^{3D}} \end{array}$$ (1)

where *f*_*β*_ is the fraction of the cells observed in a cross section of the system that are in subpopulation *β*. The value of *f*_*β*_ is affected not only by the relative frequency of the cell subpopulation *β* in the 3D tissue, but also from the increased number of intersections of the imaging plane with large or elongated 3D cells than with small or compact 3D cells. It can be connected to the mean 2D cross-sectional area of cells of that subpopulation, 〈*a*〉_*β*_, as well as to the fraction of the total cell volume in the tissue from subpopulation, $f_{\beta }^{V}$. In terms of these quantities, it is

$$\begin{array}{c} f_{\beta }=\frac{f_{\beta }^{V}/{\left\langle a\right\rangle }_{\beta }}{\sum_{\alpha }f_{\alpha }^{V}/{\left\langle a\right\rangle }_{\alpha }} \end{array}$$ (2)

where the sum occurs over each constituent cell subpopulation *α*.

Since the 2D shape distribution from heterogeneous systems is approximately given by the superposition of pure-*p*^3*D*^ distributions, the sensitivity to *p*^3*D*^-heterogeneity is linked to the variation with *p*^3*D*^ itself. The lack of change of the distribution with heterogeneity simply arises from two facts. First, many similar constituent distributions underlie the distribution of the heterogeneous system, so for instance, Gaussian-distributed *p*^3*D*^ heterogeneity with $\sigma_{p^{3D}}=0.15$, 68% of the distribution weight comes from the narrow range $\mu_{p^{3D}}-0.15<p^{3D}<\mu_{p^{3D}}+0.15$. Second, the smooth variation of the distribution with *p*^3*D*^ (*e*.*g*. Fig 2) means that Gaussian heterogeneity (which is symmetric above and below the mean *p*^3*D*^) produces somewhat canceling differences to the homogeneous distribution.

We also show explicitly in an example that variations in volume have even less impact on the estimations of cell shape. This can be seen by comparing (Fig 6(b)) distributions from a homogeneous case, *σ*_*V*_ = 0 (solid black), a *p*^3*D*^-heterogeneous case (dashed blue), and a *V*-heterogeneous case (dotted red). The mean *V* and *p*^3*D*^ are the same in all cases, and either *V* is the same for all cells while *p*^3*D*^ is drawn from a Gaussian distribution (standard deviation $\sigma_{p^{3D}}=0.15$) or *p*^3*D*^ is the same for all cells and *V* is drawn from a Gaussian distribution (*σ*_*V*_ = 0.15*μ*_*V*_).

**Fig 7:**
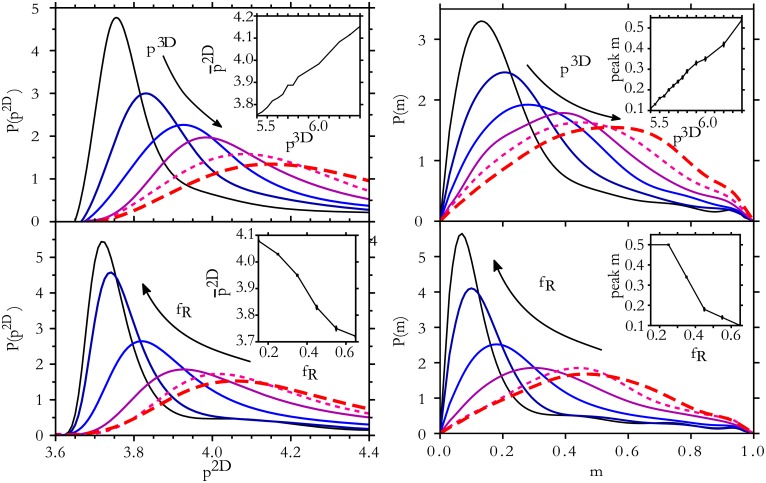
Distributions from alternative shape descriptors are similarly distinct. The distribution of *p*^2*D*^ values depends on the volume fraction of the largest inscribed sphere *f*_*R*_, and distributions of the 2D anisotropy index *m* reflect the 3D shape descriptors *p*^3*D*^ and *f*_*R*_. Curves correspond to *f*_*R*_ value, varying in 0.1 increments from 0.15 (dashed, red) to 0.65 (solid, black) or *p*^3*D*^ value, varying from 5.4 (solid black) to 6.4 (dashed red) in increments of 0.2.

The curve corresponding to heterogeneous volume is much more similar to the homogeneous case than the one corresponding to the similarly heterogeneous distribution of cell shapes. The K-S distance quantifies this; the distance between the dotted red curve and the black curve is $dD/d\sigma_{p^{3D}}\approx 0.1$, which is about 10% of the distance between the black curve and the dashed blue curve. Practically speaking, this means that the 2D shape data presented in this manuscript Figs 2, 5 and 7 provide accurate estimates of 3D cell shape even if there are fluctuations in preferred cell volume.

Taken together, these results suggest that 2D shape analyses are very good at estimating the *mean* 3D shape index, even with heterogeneity, but are not very useful for estimating the variance of the 3D shape index. Although the *p*^2*D*^-distributions vary with heterogeneity (and so the method is sensitive to heterogeneity in principle), the variation is relatively small. Future modeling work should therefore focus on understanding what features of heterogeneous systems are important for understanding global tissue mechanics.

### Alternative geometry descriptors

The above analysis focuses on cell shape indices *p*^2*D*^ and *p*^3*D*^ as 2D and 3D shape descriptors, respectively. However, the method is quite general and other shape descriptors can also be considered. Here we demonstrate this point with two other quantities. First we show that the *p*^2*D*^ distribution can be used to extract the mean value of *f*_*R*_, the ratio of the max inscribed sphere volume to Voronoi cell volume, which provides another characterization of a 3D shape. To study the distribution of *p*^2*D*^ values, we use the same set of simulations of 20,000 cells as before and bin cells by their *f*_*R*_ value. Fig 7 shows that the distribution of *p*^2*D*^ varies with the value of *f*_*R*_, which ranges between 0.1 and 0.7. The distributions in Fig 7 resemble those in Fig 2, because both *p*^3*D*^ and *f*_*R*_ characterize compactness of the 3D shapes.

We additionally ask if a different 2D shape descriptor can be used to extract the mean value of *p*^3*D*^. We consider distributions of *m*, the anisotropy index [34]. The shape anisotropy, *m*, is determined using the 2 × 2 moment of inertia tensor of the shape,

$$\begin{array}{c} G=\int_{area}\;\vec{v}⊗\vec{v}\;dx\;dy. \end{array}$$

Here, the integral runs over the area of the shape in the x-y plane. $\vec{v}\left(x,y\right)$ is the 2D vector from the shape center of mass. The anisotropy index, *m*, is the difference of the eigenvalues of the matrix *G* divided by their sum. Again, a confocal slice through a collection of cells of a specified *p*^3*D*^ produces a distribution of these 2D shape descriptors.

The additional distributions of *m*, for varied values of *p*^3*D*^ and *f*_*R*_, are also shown in Fig 7 As *m* remains bounded within the unit interval, the distribution lacks a long tail and may be preferable in some cases to *p*^2*D*^ as a 2D shape descriptor. The presence of the peak at low-*m* is reflective of the correlations between *m* and *p*^2*D*^.

Multi-dimensional (joint) distributions $P_{p^{3D}}(p^{2D},m,a)$, where *a* is cell 2D cross-sectional area, may also be used for more complete comparisons between the 3D model and the 2D geometry in slices. Additional 2D descriptors provide more sensitivity and points of comparison between experiment and model, whereas additional 3D descriptors provide more information about the 3D geometry.

### Shape estimation from experimental data

Here, we demonstrate our method on experimental data. While we have developed our method for 3D isotropic disordered animal tissues, segmenting these tissues in order to precisely measure *p*^3*D*^ remains a difficult task, and not surprisingly, we have indeed found no publicly available segmentation data of such tissues. In contrast, many plant cells are more readily imaged in 3D, but, conversely, they are typically ordered with a more regular structure. Here, we use segmented images of cells in the Arabidopsis plant, in data provided in Ref. [35], (labeled “Plant 1, Hour 0”), to illustrate the use of our method. Unlike the our 3D Voronoi model data set that our method is based on, these tissues are anisotropic. Despite these differences in geometry, we use the CVM-derived *p*^2*D*^ distributions provided above to estimate 3D cell shape.

We sample the 3D-segmented dataset on a cubic grid of spacing 0.66 × 0.66 × 0.74 *μm*. The marching cubes algorithm [36, 37] identifies the 3D surface of each cell. The mean *p*^3*D*^ is 5.5 and standard deviation is 0.2. Cells are then sorted according to *p*^3*D*^ into bins of width 0.1. For each *p*^3*D*^-bin, we slice all cells within the bin and find the *p*^2*D*^ values of shapes in the 2D slices. The K-S test indicates the most similar distribution.

**Fig 8:**
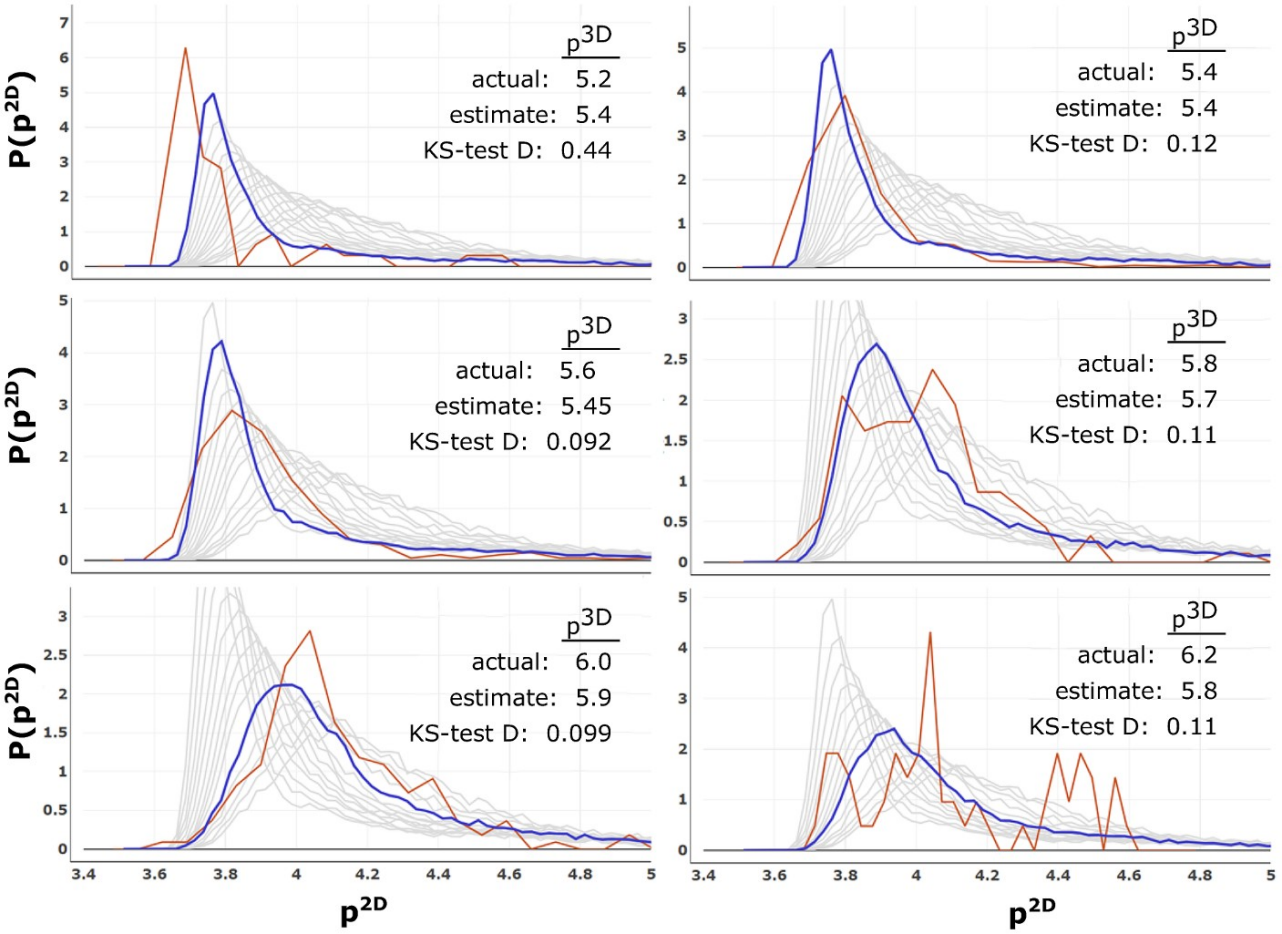
Analysis from experimental geometries. The *p*^2*D*^-distribution from slices of cell geometries extracted from experimental imagery (red) are compared with the reference distributions generated from the 3D Voronoi model (gray). The closest reference distribution (blue), identified by the K-S test, gives the *p*^3*D*^ estimate.

Fig 8 compares distributions of *p*^2*D*^ from the plant cells with those from the CVM. Although the roots are not the isotropic aggregates represented by the model, the distributions for a given *p*^3*D*^ are quite similar to the ones derived from the CVM. The estimate $p_{est}^{3D}$ is near the actual value of *p*^3*D*^. Over most of the range of *p*^3*D*^ values, $|p^{3D}-p_{est}^{3D}|\leq 0.15$. At the low end, *p*^3*D*^ = 5.2 cells are estimated to have $p_{est}^{3D}=5.4$, since that is the lowest value of *p*^3*D*^ considered in the model geometries, producing a mis-estimate of 0.2. At the high end, *p*^3*D*^ = 6.2, there are only four cells in the dataset analyzed, producing a noisy *p*^2*D*^-distribution, and a best-fit estimate of $p_{est}^{3D}=5.8$. Additional experimental 3D cells with large *p*^3*D*^ would be required to smooth the distribution. When slices are taken from the full 3D dataset without separating cells, the result is $p_{est}^{3D}=5.45$ (K-S distance 0.07), a difference of 0.05 from the mean *p*^3*D*^ = 5.50.

Notably, the plant cells are not too dissimilar from the CVM geometry, approximately having convex polyhedral shapes. Tissues with the same geometry as the CVM may compare directly to its *p*^2*D*^ distributions. Tissues with dramatically different cell shapes may expect large K-S distances from all CVM distributions, and would require distributions from an appropriate model. Because the plant cells are reasonably well-described by the CVM, the 3D shape parameters from 2D slice data that we estimate using this method are in excellent agreement with the actual 3D shape parameters obtained from the 3D cell reconstructions.

### Connection to volume or surface area determination

This analysis has focused on determination of the average dimensionless cell shape index in confluent tissues. In certain applications, it may be desired to determine properties of cell volume or surface area independently.

Techniques to estimate average cell volume can be comparatively simple, and there are several possibilities. First, from a 3D image, average cell volume for confluent tissues is simply the imaged volume divided by the number of cells. This requires counting cells (e.g. by counting the nuclei for mononucleated cells), which is much simpler than a 3D segmentation of cell membrane boundaries, as needed for cell shape.

Second, it is tempting to infer mean cell volume from dimension-full 2D cell geometry such as areas *a* or perimeters, in line with the method presented in this paper. Dimensional analysis suggests, 〈*V*〉 = *c*_1_〈*a*〉^3/2^, where 〈*a*〉 may can come from a simple count of confluent cells, and *c*_1_ still depends on cell shape. In the homogeneous 3D Voronoi geometry, we find that *c*_1_ varies between 1.52 and 1.85 and depends on the 3D cell shape parameter *p*^3*D*^ approximately as *c*_1_ ≈ 0.32 ⋅ *p*^3*D*^ − 0.2. We find no significant change in this relation when we introduce heterogeneity using a Gaussian-distributed *p*^3*D*^ or *V* up to standard deviation *σ*_*V*_ < 0.3*V*_0_ and $\sigma_{p^{3D}}<0.3$.

If cell volume can be determined, cell surface area can be approximately recovered from *p*^3*D*^, the dimensionless surface area. With heterogeneous shapes, because the estimate follows the mean *p*^3*D*^, the mean surface area is $p_{est}^{3D}V^{2/3}$—where this result is exact for homogeneous-volume cells.

## Discussion

We have investigated a method for determining statistical information about the 3D shapes of cells using only 2D slices through a tissue. We focused on the 3D shape index *p*^3*D*^ and found that it can be inferred directly and reliably from the distribution of measured *p*^2*D*^ values. We quantified the number of *p*^2*D*^ measurements (and therefore cell traces) required to reduce the random and systematic errors in *p*^3*D*^, showing that typically only of order 100 cell traces are required to reduce uncertainty in *p*^3*D*^ below a given threshold. The method is reasonably robust against the modeled sources of error and we suggest that the peak and width of the *p*^2*D*^ distribution are easily-accessible quantities that biologists can compare directly to our reference distributions [33]. We also study the effect of tissue heterogeneity, and find that 2D shapes can be used to estimate the average 3D shape, but provide little information about the variance in 3D shape.

It is possible to envision many extensions of this general framework. For example, confocal images occasionally have rather limited resolution along the z-axis, so that the effective thickness of the 2D slice is significant compared to the diameter of the cell. If the slice thickness is well-characterized, it is possible to use ray-tracing as discussed in the methods section to generate reference distributions relating 2D shapes to 3D shapes corresponding to a specific value of the slice thickness. Another extension would be to study different models for cellular structure, such as vertex models [6, 7, 38] where the degrees of freedom are cell junctions instead of the cell centers, cellular Potts models [39, 40] where each cell is composed of a grid of points, or even models for non-biological materials such as foams [41].

We note that we are assuming that the tissue is isotropic on average. For such tissues, the observed distribution of 2D shapes does not depend on the orientation of the 2D slice. Conversely, for anisotropic tissues, a dependence on orientation will generally be observed, which could be used to infer the degree of alignment. We therefore presume that it is not an experimental challenge to determine whether a tissue is anisotropic. If it is not possible to study the dependence on orientation, then tissue anisotropy will show up in the 2D shape distributions, which will typically differ from those presented here.

We further note that while it has been found that Voronoi tessellations approximate 2D tissue geometry reasonably well for many purposes [42], the degree to which the 3D Voronoi model mimics a specific 3D biological tissue can be expected to vary with the type of tissue. On the other hand, because the high sensitivity of the *p*^2*D*^ distribution to *p*^3*D*^ originates from the fact that more compact cells produce more compact cross-sections, we expect the *p*^3*D*^ estimate to be robust to minor variations in the geometry. Indeed, this is seen in the demonstration on experimental cell geometry for *Arabidopsis*, even though the tissue is anisotropic and is presumably not well-described by the cell vertex model.

It is also possible to envision extensions of this work beyond confluent cellular structures. For example, it may be possible to perform a similar analysis on particulate models and compare to nuclei-labeled images. Alternatively, cell migration in fiber networks is conjectured to be limited by the rate at which cells can squeeze their nuclei through the pores in the mesh [43]. It would be interesting to see if one could estimate typical pore sizes from statistics of 2D slices of fiber network models.

Although great progress is being made in developing techniques to fully reconstruct 3D cell shapes [22–25], their use is restricted to specimens that are optically transparent, or are compatible with light-sheet microscopy, and they can not be used in situations where cells exchange neighbors faster than a 3D scan can be completed. In contrast, 2D images of cell structure are ubiquitous in medicine and biology, from histological sections of cancer tumors for use by pathologists to standard brightfield microscopy techniques, and they are also fast to obtain.

Our results suggest that the techniques described here could be easily used by biologists and clinicians to obtain information about 3D shapes in many cases where full 3D reconstruction is not possible or extremely time-consuming. We have shown that our methods are quite robust, and that the distributions developed here work quite well even on cell shapes in an *Arabidopsis* tissue. In addition, this paper highlights a different way of thinking about how to use 2D cell images. Currently, cell shape and shape polarization are often described in terms of simple 2D quantities such as the axes of the best-fit ellipse or the length and width, and this naturally leads to a focus on how those quantities correlate with motion in a 2D plane. For cells in 3D tissue, our work suggests that 2D quantities provide information about 3D shape, which could be used to drive and test hypotheses about cell migration and tissue mechanics in 3D.

## Methods

To investigate the connection between 3D cell shape and 2D cross-sectional cell shapes in a 3D geometry that is representative of simple isotropic 3D tissues, we use the recently developed 3D Voronoi model [20]. Based on a Voronoi tessellation of cell center positions [44], we divide a simulation volume with periodic boundaries into polyhedra, representing cells. Cell centers move to minimize a Hamiltonian,

$$\begin{array}{c} E=k_{S}\sum_{i=1}^{N}{\left(S_{i}-S_{0i}\right)}^{2}+k_{V}\sum_{i=1}^{N}{\left(V_{i}-V_{0i}\right)}^{2}\text{,} \end{array}$$

in which each cell *i* has a target volume *V*_0*i*_ and surface area *S*_0*i*_, and *N* is the number of cells. We choose the target cell parameters either to be equal for all cells (*S*_0*i*_ = *S*_0_ and *V*_0*i*_ = *V*_0_) to simulate a homogeneous system, or such that target volumes *V*_0*i*_ and target shape indices $p_{0i}^{3D}=S_{0i}/V_{0i}^{2/3}$ are independently drawn from Gaussian distributions. The parameters *k*_*S*_ and *k*_*V*_ are stiffness constants which we set to 1 here without loss of generality, since we will focus on cases in the fluid regime of the model [20] where all cells attain their target parameters and *E* ≈ 0. The simulation volume is fixed at *V*_*total*_ = ∑_*i*_ *V*_0*i*_. Starting from uniformly randomly distributed cell centers, we use the FIRE minimizer [45] to minimize the energy with respect to all cell positions, generating an isotropic ensemble of 3D cell shapes.

For homogeneous target shapes, we observe that when $p_{0}^{3D}≳5.41$, all cells satisfy their optimal values, *S*_*i*_ = *S*_0_ and *V*_*i*_ = *V*_0_. This allows generation of a large ensemble of disordered cells all with the same shape index, *p*^3*D*^ = *S*/*V*^2/3^. When $p_{0}=S_{0}/V_{0}^{2/3}≲5.4$, the geometry becomes pinned [20] such that the mean shape index is 〈*p*^3*D*^〉 ≈ 5.4, because it appears that disordered packings with smaller shape index do not exist [20]. For values of *p*_0_ > 6.4, multifold vertices become common and minimization algorithms face challenges [19] and so we restrict simulations to *p*_0_ ≤ 6.4. We created packings with shape indices *p*^3*D*^ from 5.4 to 6.4 with 0.05 increments.

Once 3D packings with a defined distribution of *p*^3*D*^ are generated, we simulate the acquisition of 2D cross sections by generating images of intersections of cells with a specified plane (Fig 1) using the software POV-Ray [46]. Based on a segmentation of these cellular cross sections, we quantify the cell 2D shape index *p*^2*D*^ as the quotient of perimeter divided by the square root of the cross-sectional area, which provides us with a histogram of 2D shape indices *p*^2*D*^ for the given ensemble of cell packings with its predefined *p*^3*D*^ distribution.

To compare different *p*^2*D*^ distributions with each other, we use two kinds of measures. As a practical measure for experimentalists to extract the 3D shape index from a *p*^2*D*^ distribution, we propose to use peak and HWHM of the *p*^2*D*^ distribution (Figs 1 and 5). As a complementary measure to compare *p*^2*D*^ distributions in more detail, we also use the K-S test, which measures the maximum distance between the two respective cumulative distributions [47] (used for Figs 3–5 and 8).

### K-S test for goodness of fit and sensitivity

The Kolmogorov-Smirnov (K-S) test characterizes the likelihood of a set of measurements, given a probability distribution for the measured quantity. [47] For our purposes, K-S assigns a distance, *D*, to the dissimilarity of a measured histogram and one of the reference distribution, *P*(*p*^2*D*^). Specifically, *D* is computed from the maximum separation between the empirical cumulative distribution function (EDF) of the data and the cumulative distribution function (CDF) of the reference distribution. The CDF of a distribution *P*(*X*) is $C\left(x\right)=\int_{-\infty }^{x}dx^{\prime }P\left(x^{\prime }\right)$. The corresponding quantity for the set of *p*^2*D*^ values, {*X*_*i*_}, that go into the histogram is the EDF, $C_{\text{emp}}\left(x\right)=\frac{1}{N}\sum_{i=1}^{N}\Theta \left(x-X_{i}\right)$. *N* is the number of samples in the histogram, and Θ(*x* − *X*_*i*_) is the Heaviside step function, equal to 0.0 for *x* less than *X*_*i*_ and otherwise equal to 1.0. The K-S distance *D* is the maximum separation between the two, $D={\text{max}}_{x}\;\left|C_{\text{emp}}\left(x\right)-C\left(x\right)\right|$. This distance allows identification of the model distribution that best fits the sampled data in the main text.

The K-S test quantifies the goodness of fit between data and model in the presence of random noise [47]. This is done by comparing the K-S statistic $\sqrt{N}D$ with a published table and indicates in our context whether the 2D geometry from the experiment is consistent with the 3D geometry of the model considered.

Furthermore, the K-S distance *D* can be used to quantify the sensitivity of a distribution to *p*^3*D*^ by characterizing how quickly a *p*^2*D*^-distribution changes upon changing *p*^3*D*^. For a small change of *p*^3*D*^ by *δ*, we find that the K-S distance *D* increases from zero linearly. The sensitivity is given by *dD*/*dδ*. The *p*^2*D*^-distributions of Fig 2 are most sensitive to *p*^3*D*^ at *p*^3*D*^ ≈ 5.4 where *dD*/*dδ* ≈ 1.5, while near 6.4 the distributions are less than half as sensitive with *dD*/*dδ* ≈ 0.5. Thus, while the location of the distribution peak ${\bar{p}}^{2D}$ varies linearly with *p*^3*D*^ with slope 0.4, the changes in *distribution shape* produce additional sensitivity near *p*^3*D*^ = 5.4.

Similarly, the sensitivity of the *m*-distribution to *p*^3*D*^ is found to be $dD_{p^{3D},m}/d\delta \approx 1.4$ at *p*^3*D*^ = 5.4 and falls to $dD_{p^{3D},m}/d\delta \approx 0.4$ by *p*^3*D*^ = 6.4. Thus distributions of *m* and *p*^2*D*^ are about equally sensitive to *p*^3*D*^, and the uncertainties in Figs 3 and 4 in the main text would be similar for *m*. Here $D_{p^{3D},m}$ is the K-S distance between *m*-distributions from *p*^3*D*^ values that differ by *δ*. Analogously, $D_{f_{R},p^{2D}}$ being the K-S distance between *p*^2*D*^-distributions from *f*_*R*_ values that differ by $\delta_{f_{R}}$, the sensitivity to *f*_*R*_ is found to be $dD_{f_{R},p^{2D}}/d\delta_{f_{R}}\approx 0.5$ and $dD_{f_{R},m}/d\delta_{f_{R}}\approx 0.6$ near *f*_*R*_ = 0.2, rising to $dD_{f_{R},p^{2D}}/d\delta_{f_{R}}\approx 2.7$ and $dD_{f_{R},m}/d\delta_{f_{R}}\approx 2.3$ at *f*_*R*_ = 0.5. This quantifies the level of sensitivity that is evident in Fig 7.
